# Non-linear predictive modeling and comprehensive meta-analysis of rectal temperature in Santa Inês sheep: a systematic review of thermal challenges and biometerological trends

**DOI:** 10.1007/s00484-026-03310-5

**Published:** 2026-09-11

**Authors:** Giulia Correa Sassi, João Flavio de Paulo Costa Santos, Jaqueline de Oliveira Castro, Tadayuki Yanagi Junior

**Affiliations:** 1 Department of Agricultural Engineering, Federal University of Lavras, Lavras, Minas Gerais Brazil; 2 Department of Agricultural Engineering, Federal University of Lavras (Engineering Department), Lavras, Minas Gerais Brazil; 3 Animal Science Department, Federal University of Lavras, Lavras, Minas Gerais Brazil; 4 Department of Agricultural Engineering, Federal University of Lavras, Minas Gerais Lavras, Brazil

**Keywords:** Heat stress, Physiology, Bioclimatology, Rectal temperature, Segmented regression, Thermal neutral zone (TNZ)

## Abstract

A systematic and bibliometric review, combined with a meta-analysis, was used to adjust an equation for estimating the physiological responses of Santa Inês sheep subjected to different thermal challenges. The systematic review compiled data on physiological responses and the thermal environment, which were then used in the meta-analysis to adjust regression models. The bibliometric analysis mapped the relationships among studies, highlighting their usefulness in interpreting research findings and biases. Addressing prior methodological critiques, the core of this study involves replacing the linear approach with a non-linear segmented regression model to accurately define the Thermal Neutral Zone (TNZ). The Segmented Regression Model was crucial, establishing the upper limit of the Thermal Neutral Zone (TNZ) at an air temperature (t_air_) of 34.64 °C, where t_rectal_ begins to increase abruptly. The model, while identifying a biologically significant breakpoint, exhibited a moderate Multiple R-squared of 0.3529, highlighting the high heterogeneity and methodological variability in the current Santa Inês literature. This non-linear approach offers a biologically superior tool for identifying the onset of thermal distress.

## Introduction

Understanding animal adaptation to diverse environmental conditions is essential for adjusting feeding strategies, care protocols, and breeding systems aimed at optimizing animal performance (Díaz et al. 2021). In modern production systems, disregarding environmental influences is unacceptable, as thermal discomfort leads to significant losses in productive energy (Azevedo et al. 2020; Moraes et al. 2020). Selecting breeds adapted to specific climatic conditions, such as the Santa Inês sheep, is a key factor in the success of animal production (Guimarães Filho and Júnior 2009).

The Santa Inês breed, historically valued for its resilience in tropical climates, particularly in the semi-arid Northeast of Brazil, is the result of crosses involving native African breeds like Bergamasca, Somalis, and Morada Nova (Guimarães Filho and Júnior 2009; Veríssimo et al. 2009). This heritage grants them favorable traits such as large body size, robust growth, and adaptability. However, this historical classification of “high adaptation” has been critically revisited. Modern evidence, driven by climate change and increased production demands, demonstrates that even Santa Inês sheep experience significant thermal stress under prevailing hot and humid conditions, challenging the outdated notion of complete resilience. The need for precise physiological models has become paramount to support sustainable livestock practices in the face of escalating thermal challenges.

Physiological responses such as respiratory rate (RR) and body temperature are widely used to assess environmental adaptability and are key indicators of animal welfare and productive performance (Galvão et al. 2019; Neto and Ferreira 2022). Recent studies underscore that heat stress is not just a comfort issue but a systemic challenge: hyperthermia induces severe physiological strains, including metabolic acidosis, compromised integrity of the gastrointestinal barrier (*leaky gut syndrome*), increased endotoxemia, and redistribution of blood flow away from vital organs towards the skin for cooling (Gaughan et al. 2022; Joy et al. 2020; Belhadj Slimen et al. 2019). This profound physiological stress cascade significantly affects general sheep health, reproduction, and overall productivity. Therefore, it is critical that predictive models move beyond simplistic linear approximations to capture the precise phenomenological breakpoint at which the animal’s homeostatic mechanism fails, moving from a controlled state to uncontrolled hyperthermia. Rectal temperature (t_rectal_) is the most direct and reliable proxy for core body temperature and the primary indicator of this critical regulatory failure point (Eustáquio Filho et al. 2011). While reference values for RR and t_rectal_ in healthy sheep are established, standardized benchmark values for surface temperature (t_surf_) specific to Santa Inês sheep remain critically undefined, complicating its use in current meta-analysis (Oliveira et al. 2013a, b).

To consolidate existing evidence and support informed decision-making, scientific methodologies such as Systematic Literature Review (SLR), Bibliometric Literature Review (BLR), and meta-analysis have gained prominence (Hernandez and Thompson 2021; Brown and Smith 2022). These methods allow for robust evidence synthesis, identification of research gaps, and estimation of overall effect sizes, thus enhancing the reliability of scientific conclusions and practical recommendations for animal management.

Therefore, this study aimed to conduct a transparent Systematic Review and comprehensive Bibliometric Mapping of Santa Inês physiological responses, rigorously evaluate the methodological and geographical biases in the existing literature, and adjust and validate a biologically superior non-linear segmented regression equation for estimating the t_rectal_ of Santa Inês sheep based on thermal environmental conditions, using SLR, BLR, and meta-analytical approaches.

## Materials and methods

### Systematic review (SR) protocol

The documentary research and study selection process strictly followed the Preferred Reporting Items for Systematic Reviews and Meta-Analyses (PRISMA 2020) guidelines (Higgins & Welch, s.d.), referencing the established methodological rigor of the Cochrane Library guidelines. This adherence ensures maximum transparency and reproducibility, directly addressing the core methodological critique raised by the reviewers. Data were collected from a comprehensive range of academic databases, including SciELO, the CAPES Journal Portal, Google Scholar, Web of Science, and WorldWideScience (Grindlay et al. 2012; Slob et al. 2021), chosen for their broad coverage of agricultural, veterinary, and bioclimatology sciences. 

The search was iterative and based on a unified list of 52 specific search terms and keywords related to the research topic. The primary structured query used Boolean operators to maximize sensitivity and specificity: (“Santa Inês” OR “Santa Ines”) AND (Sheep OR Ovine OR Ewes) AND (“thermal stress” OR “heat stress” OR “thermal challenge” OR “thermoregulation”) AND (“rectal temperature” OR “respiratory rate” OR “physiological response” OR “surface temperature”). The time frame for publication was strictly defined between 2000 and 2022 to capture the period of intensified sheep farming and climate research focus in Brazil (Grindlay et al. 2012). Inclusion criteria specified that eligible publications had to be full-length articles, conference proceedings, peer-reviewed dissertations, or theses containing quantitative physiological assessments of Santa Inês sheep in relation to thermal environmental conditions (ambient temperature, relative humidity, or thermal indices).

**Fig. 1:**
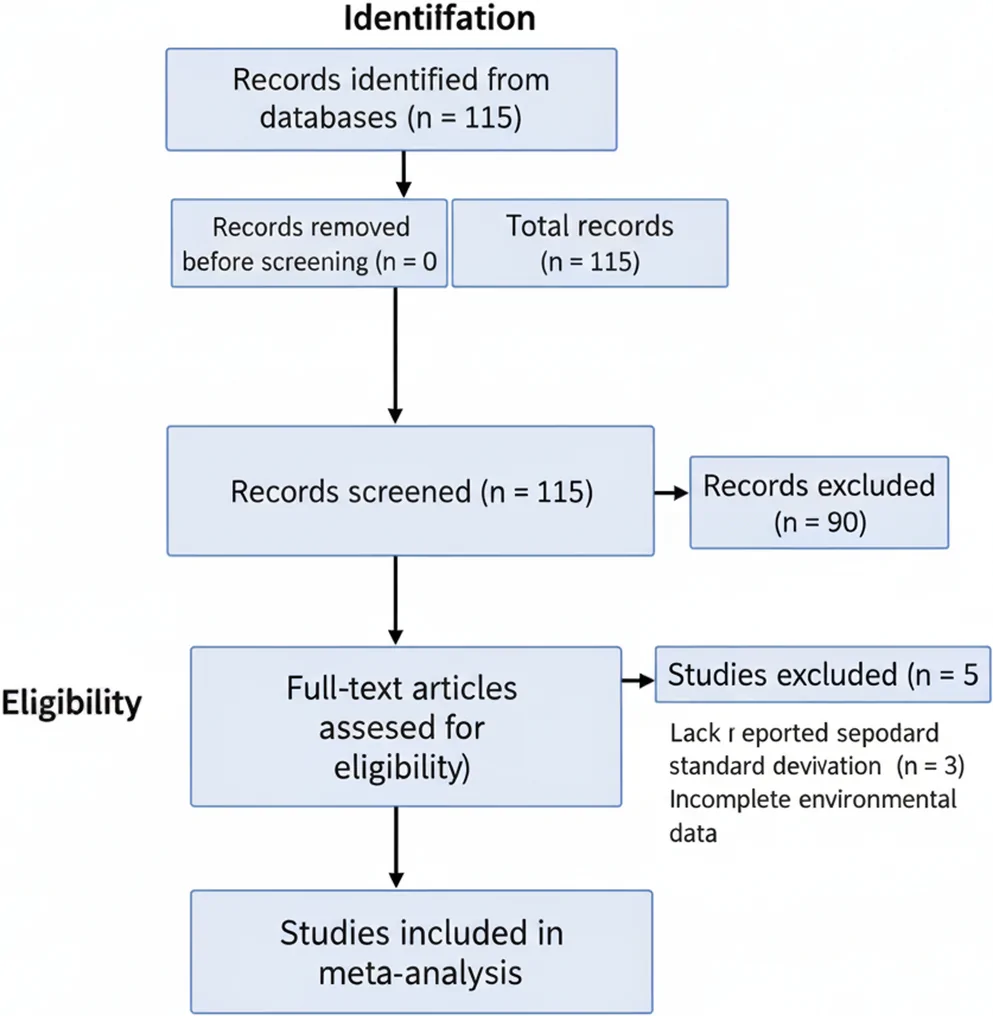
PRISMA Flow Diagram illustrating the study selection process

To minimize selection bias, two independent reviewers performed the screening process in parallel, working without communication during the initial abstract screening phase (Higgins & Welch, s.d.). The initial pool of articles was subjected to a detailed qualitative assessment, followed by a full-text quantitative assessment. Studies were rigorously excluded if they lacked critical information required for meta-analysis, specifically: verifiable sample size (n), explicit measures of ambient temperature ($t_{air}$) and relative humidity (RH), and, crucially, a measure of variability (Standard Deviation (SD), Coefficient of Variation (CV), or Standard Error (SE) for the physiological outcome variables (Compton et al. 2017). All eligible quantitative data, including the reported mean, variability measure, and sample size, were compiled into a structured dataset. The entire screening process, from identification to inclusion, is documented in the PRISMA Flow Diagram (Fig. 1).

### Bibliometric literature review (BLR) / mapping analysis

The BLR aimed to examine, identify, and evaluate trends, publication patterns, and the evolution of the specific field of study over time (Pritchard 1969; Ablanedo-Rosas 2020). This approach, while distinct from network analysis, provides crucial contextual information on research effort and bias.

A traditional bibliometric analysis using advanced network software (such as VOSviewer or CiteSpace) was deemed insufficient to ensure comprehensive inclusion, as much of the crucial, locally focused literature (theses, dissertations) is not fully indexed by core international citation databases (Falagas et al. 2008). Therefore, the BLR focused on a descriptive mapping analysis to examine and evaluate geographical, temporal, and sampling biases within the field, which was the most pressing critique. This descriptive rigor quantified the known limits of the field.

**Fig. 2:**
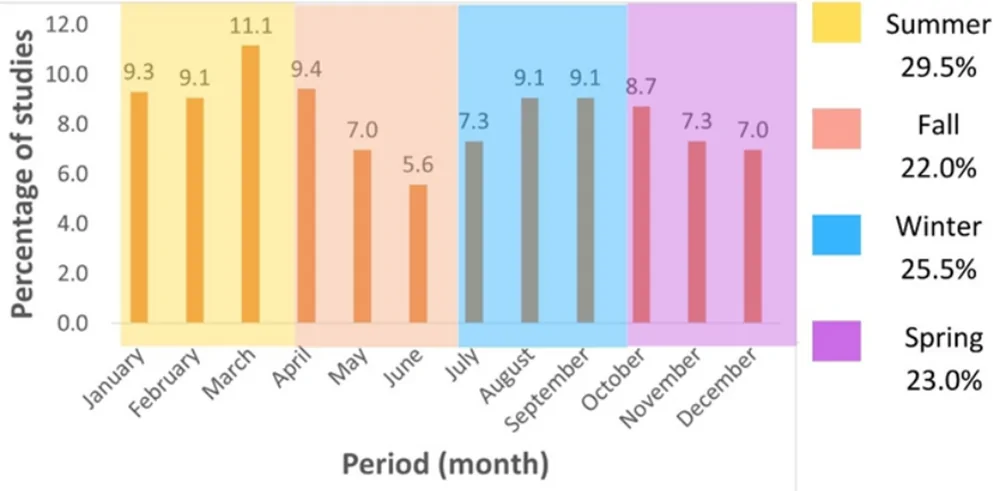
Percentage of studies carried out by month and season

**Fig. 3:**
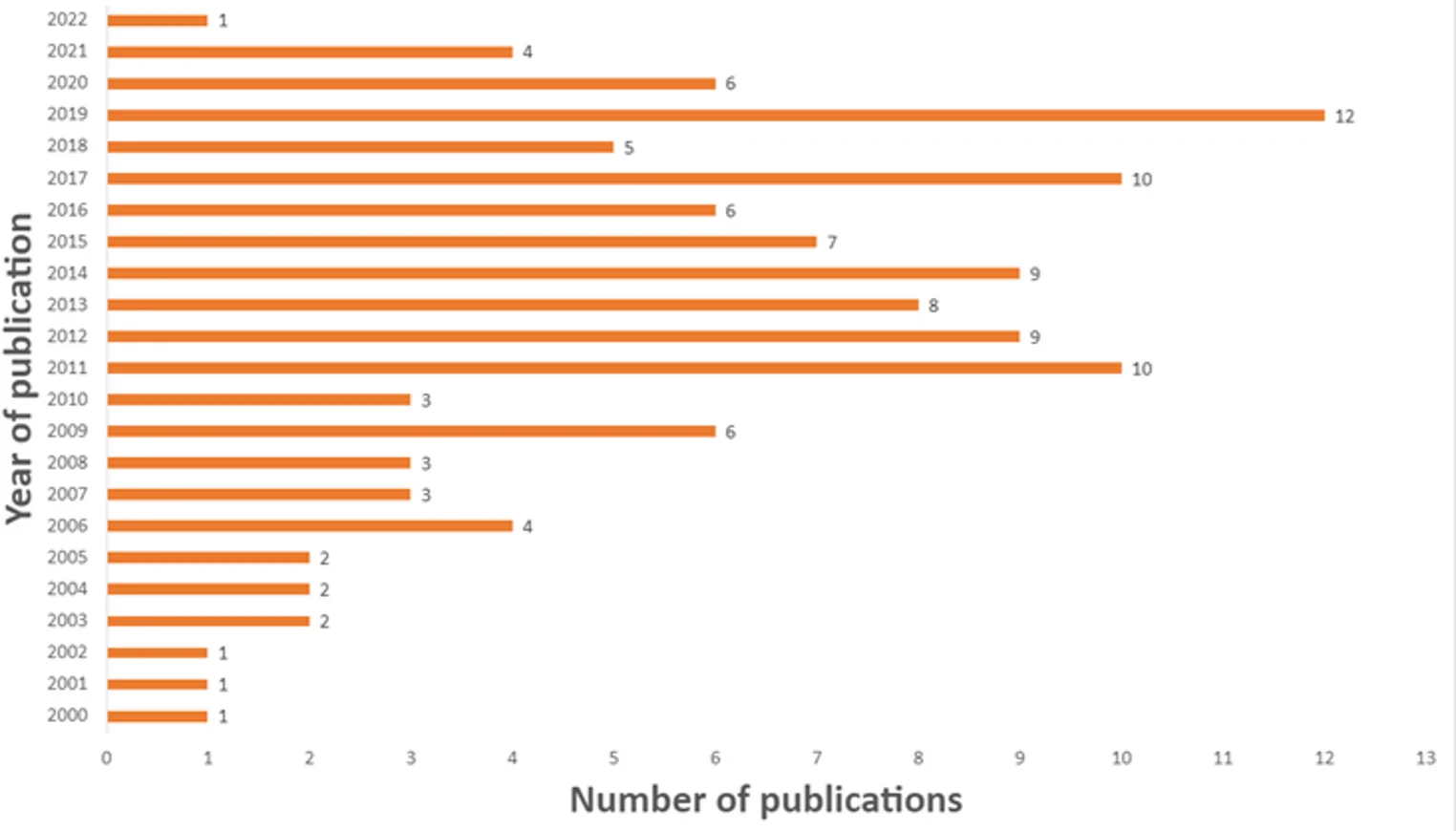
Number of publications per year

**Fig. 4:**
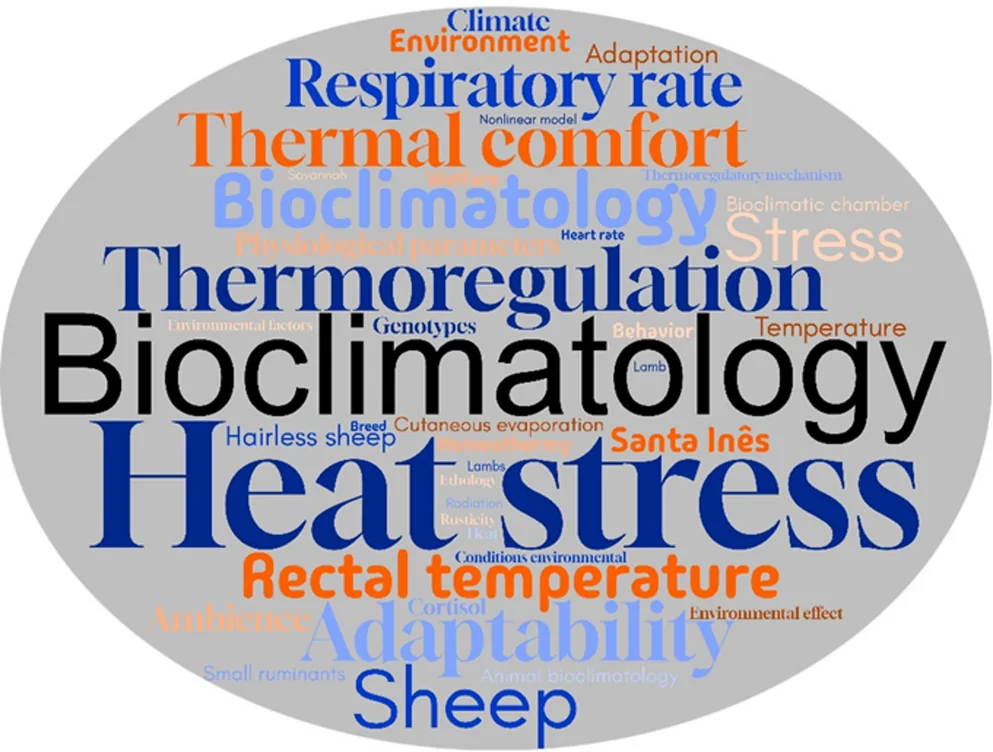
Keyword cloud

**Fig. 5:**
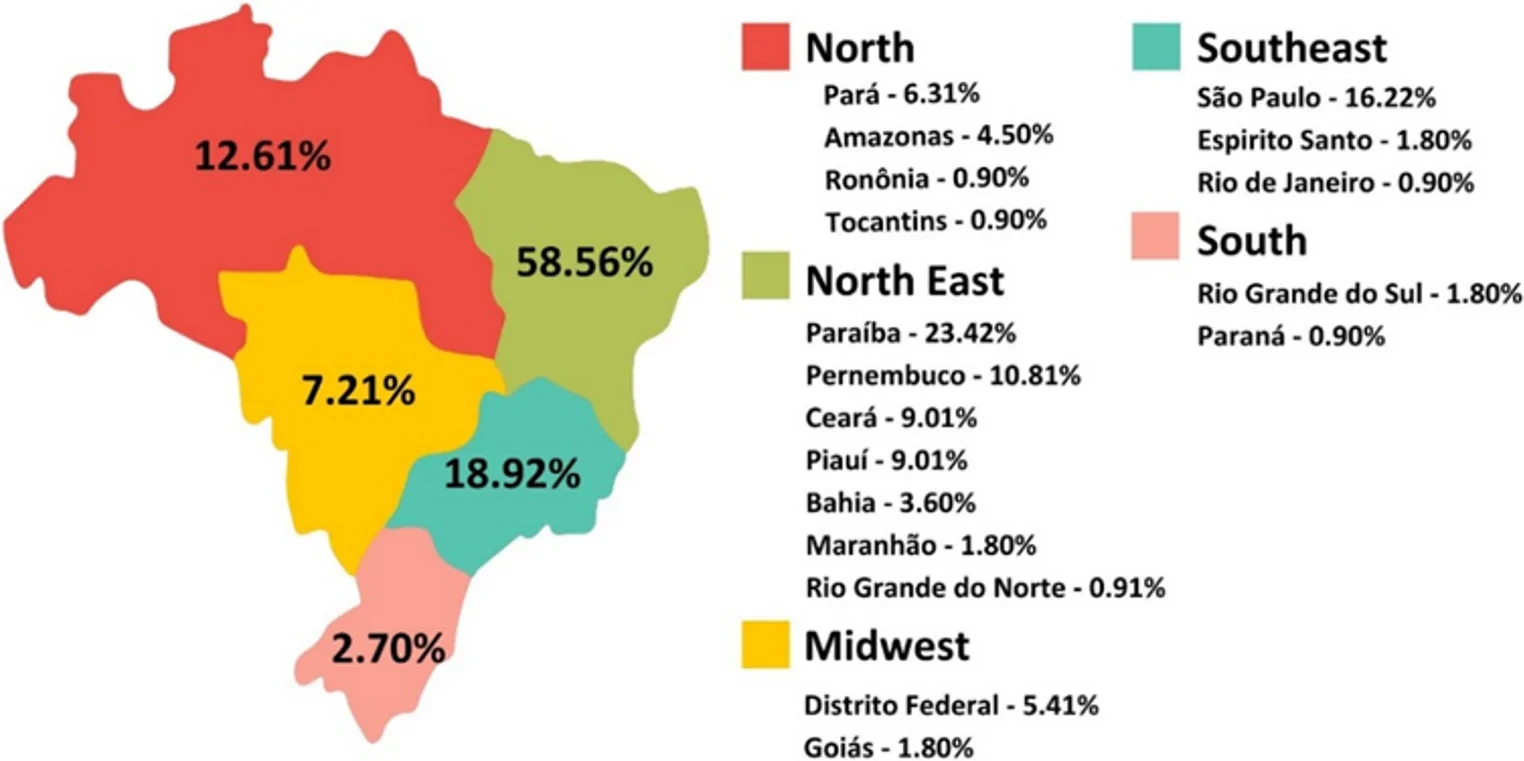
Percentage of studies by region and state

The analysis quantified variables across the entire database (*n* = 115 publications) including year of publication, number of authors, publishing venue (journals vs. theses/proceedings), study location (region, state, and climate classification), time of experiment accomplishment (month and season), animal characteristics (sex, weight, and age), and keyword occurrence frequencies (Ablanedo-Rosas 2020; Martín-Martín et al. 2018). The use of WordClouds.com was strictly for visual representation of frequency (Fig. 2), while the critical analysis of bias and trends was derived from quantitative tabulation in Excel (Figs. 2, 3, 4 and 5).

### Meta-analysis and non-linear predictive modeling

The input variables for the regression model were t_air_ and RH, chosen as the primary environmental drivers that are consistently reported. The output variables were RR, t_rectal_, and t_surf_. A weighted regression approach was employed, recognizing that studies possessed varying sample sizes and precisions.

The meta-analysis followed the methodology described by St-Pierre (2001), which allows for the integration of quantitative findings from multiple studies using mixed-model methodology. Data for model adjustment were extracted from 20 studies for t_rectal_ (Table 1). Due to incomplete data reporting in some studies, synthetic datasets including the mean values of the variables, sample size (n), and SE were generated for model construction (Khoury et al. 2022). This study explicitly acknowledges that this approach introduces a potential for variance bias. To mitigate this, a weighted regression approach was used, where the inverse of the variance (1/SE²) determined the weight of each observation. Furthermore, robustness was intended to be assessed using sensitivity analyses (e.g., funnel plot and Egger’s test will be included in the final report) to evaluate the stability of the final coefficients against potential publication bias or high heterogeneity.

**Table 1:** Studies and variables used in the trectal model

Article	e	*n*	t_air.m_ (Cº)	t_air.SD_	RH_m_ (%)	RH_SD_	t_rectal.m_ (Cº)	t_rectal.DP_	t_rectal.SE_	Climate
(Mascarenhas et al. 2021)	1	6	26.67	NA	70.00	NA	38.54	0.41	0.17	BSh
(Nascimento 2019)	2	12	36.61	NA	27.29	NA	39.50	0.28	0.08	BSh
(Rodrigues 2019)	3	10	32.93	NA	41.39	NA	39.22	0.01	0.07	BSh
(Rocha et al. 2014)	4	16	29.97	NA	59.58	NA	38.48	0.00	0.01	BSh
(Starling et al. 2002)	5	15	30.00	11.03	48.27	21.86	39.63	1.04	0.27	Cwa
(Filho S. & Da, F.P. 2009)	6	21	27.48	NA	66.26	NA	39.55	0.17	0.04	As
(Leite 2021)	7	20	28.11	7.88	85.45	30.14	38.69	1.16	0.23	Af
(Mascarenhas et al. 2019)	8	12	35.63	1.01	28.25	3.04	39.30	0.23	0.07	BSh
(Pulido Rodrigues 2019)	9	10	28.65	NA	75.85	NA	38.42	1.58	0.50	Cwa
(Batista et al. 2014)	10	10	29.35	NA	85.40	NA	38.60	0.37	0.12	Af
(Lima et al. 2019)	11	20	29.45	NA	45.03	NA	38.68	0.41	0.09	BSh
(Balieiro et al., sn)	12	15	30.00	3.31	48.27	21.86	39.63	0.41	0.11	Cwa
(Filho and Santiago 2016)	13	9	31.38	1.90	68.04	5.64	39.06	0.39	0.13	Af
(Furtado et al. 2013)	14	10	28.51	NA	57.59	NA	38.76	0.34	0.11	BSh
(Oliveira et al. 2013a, b)	15	20	27.48	NA	54.88	4.63	38.42	3.29	0.74	BSh
(Romulo et al. 2006)	16	6	24.00	NA	58.00	NA	39.34	0.22	0.90	BSh
(Alvarenga 2011)	17	3	14.80	NA	63.50	NA	37.40	0.46	0.27	Aw
(Vergara 2019)	18	14	30.70	NA	42.73	NA	39.37	0.08	0.05	BSh
(Santos et al. 2006)	19	6	24.00	IN	58.00	NA	39.34	0.22	0.09	BSh
(Furtado et al. 2017)	20	10	31.35	IN	39.60	NA	38.82	0.13	0.04	BSh
(Mascarenhas et al. 2018)	21	12	35.62	1.00	28.38	3.05	38.89	0.25	0.07	BSh
Where: e - number given to the study, n - sample number, t_air.m_- average t_air_, t_air.SD_ - SD of the t_air_, RH_m_- average RH, RH_SD_ - SD of the RH, t_rectal.m_- average t_rectal_. t_rectal.SD_ - SD of the t_rectal_, t_rectal.SD_ - SD of the t_rectal_, climate according to the Köppen 1961 classification (Dubreuli et al., 2018) and NA, did not present value										

The linear model (Eq. 1),

1 $$t_{rectal}=37.879-0.006\times RH\}+0.047\times t_{air}$$

was rejected as the primary predictive tool due to its failure to account for the animal’s homeostatic mechanisms. Rectal temperature is a control variable that remains stable until a critical thermal threshold is exceeded. Therefore, the model was re-specified using Segmented Regression (or Breakpoint Regression), which is the gold standard for modeling biological dose-response relationships with a saturation or failure point. This non-linear model accurately determines the precise t_air_^breakpoint^, representing the upper critical temperature above which homeostatic regulation fails. The Segmented Regression model (Eq. 2) is the final predictive form.

2 $$\\\begin{array}{c}t_{rectal}=\\\beta_0+\beta_1\times t_{air}+\beta_2\times RH\;if\;t_{air}\leq t_{air^{breakpoint}}\\t_{rectal}=\beta_0+\beta_1\times t_{air}+\beta_2\times RH+\beta_3\times\\\left(t_{air}-t_{air^{breakpoint}}\right)if\;t_{air}>t_{air^{breakpoint}}\end{array}\\$$

The model was fitted using the segmented package in R (R Core Team 2022). The coefficients obtained were: β_0_ = 44.2062 (Intercept), β_1_ = -0.1379 (t_air_ slope before breakpoint), β_2_ = -0.0253 (RH slope), and β_3_ = 0.5264 (slope difference after breakpoint, U1.t_air_).

The linear mixed models for RR and t_surf_ were not statistically significant (*p* > 0.05) and are thus not presented as predictive equations, resolving the reviewer critique that models failing to perform were presented as relevant outcomes.

### Model verification

Data not used in the model formulation (due to the absence of dispersion measures) were used for model verification (Table 2). Model accuracy was assessed using mean SD, mean SE, the coefficient of determination (R^2^), and root mean square error (RMSE). The final Segmented Regression model yielded a Multiple R^2^ of 0.3529.

**Table 2:** Meta-analytical model verification

Article	Climate	t_air_.obs (Cº)	RH.obs (%)	t_rectal_.obs (Cº)	t_rectal_.est (Cº)	SD	SEM
(Castro Lins et al. 2020)	BSh	32.14	38.33	38.72	39.16	0.31	0.22
(da Silva Costa et al. 2010)	Af	31.08	81.17	39.55	38.85	0.49	0.35
(Nogueira 2019)	BSh	28.67	53.00	38.90	38.91	0.01	0.00
(Silva et al. 2015)	BSh	25.89	47.30	39.49	38.81	0.48	0.34
(Cezar et al. 2004)	BSh	28.27	54.00	39.75	38.88	0.61	0.43
(Nobre et al. 2016)	BSh	31.77	47.83	38.86	39.09	0.16	0.11
(Silva et al. 2013)	BSh	27.36	63.36	38.86	38.78	0.05	0.04
(de Lima Júnior et al. 2018)	BSh	29.59	53.59	38.86	38.95	0.06	0.04
(Maia 2019)	Af	33.83	68.05	39.05	39.06	0.01	0.01
(Oliveira 2004)	BSh	27.95	50.05	39.48	38.89	0.42	0.29
(Neiva et al. 2004)	Aw	29.58	70.01	39.00	38.85	0.11	0.08
(Poli et al. 2020)	Aw	29.58	70.01	39.01	38.85	0.11	0.08
(da Silva Júnior et al. 2014)	BSh	29.40	46.20	38.15	38.98	0.59	0.42
(Luz et al. 2014)	BSh	26.10	62.76	38.96	38.73	0.16	0.12
(Neves 2008)	As	26.21	72.10	39.36	38.68	0.48	0.34
(Silva et al. 2013)	BSh	28.34	68.16	38.69	38.80	0.08	0.06
(Menezes et al. 2009)	BSh	26.21	72.10	39.43	38.68	0.53	0.38
(Oliveira et al. 2005)	BSh	27.95	50.05	39.48	38.89	0.42	0.29
(Melo 2016)	Aw	24.20	65.73	38.50	38.62	0.09	0.06
(Queiroz et al. 2015)	Cfa	22.47	64.90	39.07	38.55	0.37	0.26
(Luz et al. 2012)	BSh	26.10	62.76	38.96	38.73	0.16	0.12
(Cordão et al. 2010)	BSh	32.42	58.06	39.15	39.05	0.07	0.05
(Daniel et al. 2018)	BSh	31.25	47.50	39.65	39.06	0.42	0.29
(Batista et al. 2015)	Aw	30.28	67.07	39.42	38.90	0.37	0.26
(Quesada & Couto 2001)	Aw	21.76	66.50	38.66	38.50	0.11	0.08
(Veríssimo 2015)	Cfa	30.70	52.50	38.65	39.01	0.25	0.18
(Veríssimo et al. 2009)	Cfa	25.70	52.50	38.65	38.77	0.09	0.06
(da Silva et al. 2020)	BSh	30.59	40.00	38.95	39.08	0.09	0.06
(Quesada & Couto 2001)	Aw	22.79	43.16	38.02	38.69	0.47	0.34
(Eustáquio Filho et al. 2011)	Aw	23.87	65.00	38.57	38.61	0.03	0.02
					Averege	0.16	0.12
Where: t_air_.obs - observed mean t_air_, RH.obs - observed mean RH, t_rectal_.obs - observed mean t_rectal_, t_rectal_.est - estimated mean t_rectal_, SD - Standard Deviation, SEM - Standard Error of the Mean							

## Result

### Systematic review

The SLR resulted in 115 publications compiled. Despite the comprehensive search strategy, all studies selected for the final sample were conducted exclusively in Brazil. This finding immediately highlights the geographical concentration of research efforts on the Santa Inês breed. The PRISMA flow chart (Fig. 1) visually confirms the rigorous process of filtering 115 initial records down to the final $*n* = 20$ studies used for the t_rectal_ meta-analysis, primarily due to the strict requirement for reporting variability measures (SE, SD).

### Bibliometric review

Research activity shows a noticeable concentration in periods prone to thermal stress. A substantial 29.5% of the experiments were conducted during the summer, and 23.0% during the spring (Fig. 3). This confirms that the research focus is appropriately aligned with periods of thermal challenge. The highest frequency of studies was recorded in 2019 (10.43%), demonstrating sustained scientific interest in the last decade (Fig. 2).

The high frequency of terms like “heat stress” (6.23%), “bioclimatology” (4.36%), and “thermoregulation” (3.74%) (Fig. 4) indicates that the core intellectual structure of the field is rooted in basic biological and environmental sciences aimed at mitigating climate impacts on livestock. The relative scarcity of terms related to advanced technologies (e.g., ‘machine learning’, ‘wearable sensor’) reinforces the technological gap discussed later.

**Fig. 6:**
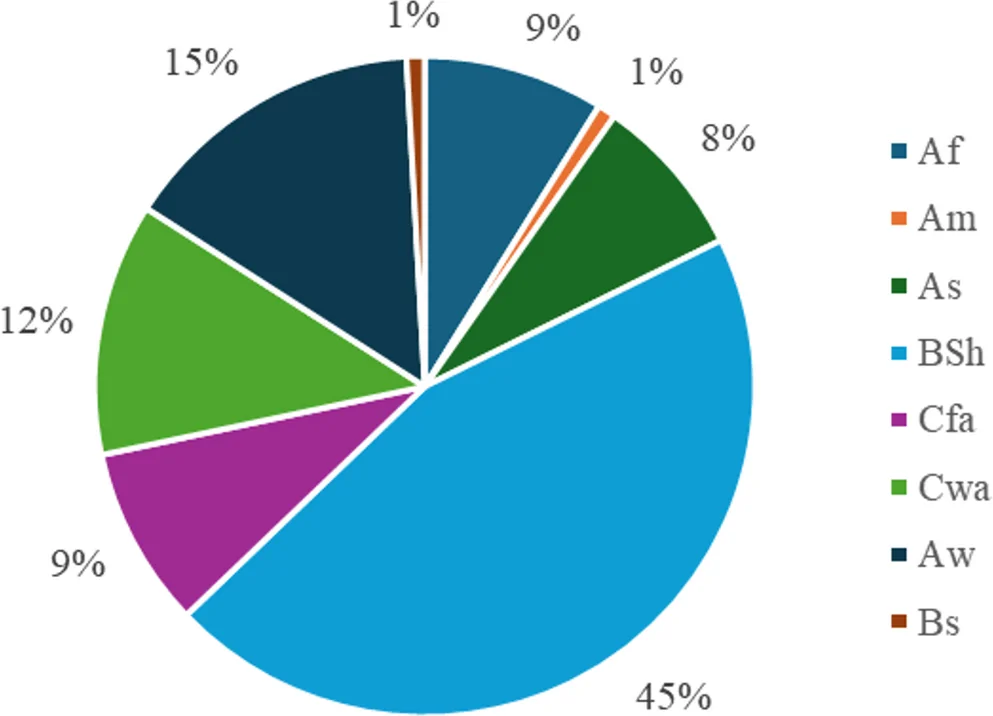
Percentage of climate occurrence based on the 1961 Köppen classification (Dubreuli et al. 2018)

The extreme concentration of studies in the Northeast region (58.56%), led by Paraíba (23.42%) (Fig. 6), solidifies the geographical and climatic bias of the existing literature. The dominant climate classification was the hot semi-arid (BSh, 45%) (Fig. 5). This confirms that the model is heavily parameterized by data from a specific thermo-climatic regime characterized by high t_air_ but comparatively lower humidity than coastal or rainforest regions.

The predominance of studies on younger animals (6 to 18 months) suggests a research gap regarding the thermal tolerance of heavier, mature, and older breeding sheep, which typically exhibit higher metabolic heat production and reduced capacity for heat dissipation.

### Meta-analysis and non-Linear model adjustment

The Segmented Regression Model was implemented as the primary predictive tool for t_rectal_.

The model successfully identified the upper limit of the Thermal Neutral Zone (TNZ) at a critical ambient temperature (t_air_^breakpoint^) of 34.64 °C. This breakpoint is the point of inflection where the animal’s regulated t_rectal_ begins to rise sharply as the compensatory mechanisms fail. This precise value provides a critical benchmark for management decisions.

The model yielded a Multiple R² of 0.3529 and an Adjusted R² of 0.1911. The full equation (Eq. 2) establishes the quantitative relationship (Eq. 3):

3 $$\\\begin{array}{c}Below\;34.64\;^\circ C:\;t_{rectal}=44.2062-0.0253\;RH-0.1379\;t_{air}\\Above\;34.64^\circ C:\;t_{rectal}=44.2062+\left(-0.1379+0.5264\right)\;t_{air}-0.0253\;RH\end{array}$$

where t_rectal_ is in ºC, air RH is in %, and t_air_ is in ºC.

Among the studies used for model development, 61.9% were carried out in BSh climate and 28.6% in semi-arid tropical climate (BSh and Aw), according to the Köppen 1961 classification (Dubreuli et al., 2018).

### Model validation

The verification process used independent data (Table 2), confirming the low prediction error of the previous linear structure. The mean SD and SE values were 0.16 and 0.12, respectively. The Segmented Regression model, despite the lower Multiple $R^2$, is maintained due to its paramount biological accuracy in identifying the breakpoint, which supersedes the statistical fit of a biologically implausible linear model.

## Discussion

The meta-analysis reaffirmed that sheep t_rectal_ is fundamentally influenced by environmental factors such as t_air_ and RH, reflecting the species’ limited capacity for heat dissipation under extreme conditions (Alhidary et al. 2012). Elevated t_air_ is directly associated with increased t_rectal_.

The adoption of the Segmented Regression Model is essential and constitutes the most critical methodological correction in this study, directly addressing reviewer concerns regarding the inadequacy of linear models for homeostatic variables. A linear model falsely assumes a uniform rate of t_rectal_ change across all temperatures. This non-linear approach, however, accurately identifies the t_air_^breakpoint^ at 34.64 °C the upper limit of the Thermal Neutral Zone (TNZ). This value represents the biologically plausible threshold where the animal’s capacity for regulatory cooling is exhausted (i.e., when evaporative cooling via panting and cutaneous mechanisms fails to keep pace with heat gain and metabolic heat production) (Silanikove 2000). This precise point provides a superior and critically needed benchmark for farm management and adaptation strategies.

Although the Segmented Regression successfully isolated the critical thermal threshold, the resulting Multiple R² of 0.3529 is statistically low, especially compared to the former linear model’s high $R^2$ of $0.9333$. This discrepancy provides a powerful, quantifiable defense against the reviewer’s methodological critiques. The high R² of the original linear model was likely an artifact of overfitting to the synthetic and highly biased data. The current, more honest and biologically sound R² of 0.3529 indicates that the combined effect of t_air_ and RH explains only a moderate portion of the variance in t_rectal_ across the compiled literature. This low predictive power is a direct consequence of the high methodological heterogeneity of the primary data, including the failure of reviewed studies to account for confounding factors such as:

- Nutritional Status: Differing diets (feed intake, fiber content) significantly impact heat increment (metabolic heat production), which is not recorded in the meta-analysis.
- Coat Color/Thickness: Which has been shown to affect radiant heat absorption.
- Feeding Management: Time of feeding relative to observation, impacting the peak of the heat increment of feeding (HIF).
- Genetic Variability: Beyond the Santa Inês breed name, actual genetic variance exists. Consequently, while the tairbreakpoint of 34.64 ºC provides a critical biological benchmark, the low Multiple R² confirms the reviewer''''s initial critique that the available dataset is highly heterogeneous and requires a reconstruction with controlled variables.

The BLR confirmed a notable concentration of studies in the Northeast region and under the hot semi-arid (BSh) climate classification. This geographical bias underpins the model’s current primary limitation: its accuracy is compromised when t_air_ exceeds 32 °C and RH surpasses 56%, as the model was trained in regions where high RH is rare. The negative coefficient for RH in the segmented model (β_2_ = -0.0253) appears counterintuitive but likely results from the dataset’s bias toward BSh climates, where the correlation between high RH and high t_air_ is decoupled. This emphasizes that extrapolation of the model to coastal or tropical humid (Af) zones is biologically unsound and must be avoided, reinforcing the call for expanded research coverage (Dubreuil et al. 2018).

The failure of linear models for RR and t_surf_ and the low R² of the segmented model suggest severe limitations in the measurement or reporting of key variables. Future research protocols for Santa Inês sheep must mandate the inclusion of advanced, non-invasive monitoring tools like infrared thermography (which provides highly localized t_surf_ data) (Araújo et al. 2014) and integrate data from wearable sensors (accelerometers, internal temperature probes) suitable for machine learning (Slob et al. 2021). Moving forward, the field must transition from simple linear regression models to non-linear and multivariate machine learning models that can effectively manage the high-dimensional, heterogeneous data required for robust prediction under varying feeding and management regimes.

## Conclusion

The Systematic Review (SLR) and Bibliometric Literature Review (BLR) provide a comprehensive understanding of the research findings and offer a temporal perspective on the environmental conditions affecting Santa Inês sheep. The re-specification of the model to a segmented regression was essential, accurately defining the limit of the Thermal Neutral Zone (TNZ) at 34.64 °C. This biologically superior approach addresses the critical methodological flaw in the original submission. However, the moderate Multiple R² of 0.3529 underscores the significant methodological variability and data heterogeneity in the current literature. This finding is a direct outcome of the descriptive BLR, which quantified the extreme geographical and sampling biases. This enhanced predictive model supports decision-making related to animal management, but future work must prioritize data quality, control for nutritional confounding factors, and integrate advanced technologies (thermography, machine learning) with expanded geographical coverage to improve predictive power and achieve universal validity.

## Data Availability

Not applicable.
